# Genetic Characterization and Phylogenetic Analysis of Small Ruminant Lentiviruses Detected in Spanish Assaf Sheep with Different Mammary Lesions

**DOI:** 10.3390/v10060315

**Published:** 2018-06-09

**Authors:** Elena Gayo, Vincenzo Cuteri, Laura Polledo, Giacomo Rossi, Juan F. García Marín, Silvia Preziuso

**Affiliations:** 1 Pathological Anatomy Section, Animal Health Department, School of Veterinary Medicine, University of Leon, via Profesor Pedro Carmenes s/n Campus de Vegazana, 24071 León, Spain; egayr@unileon.es (E.G.); jfgarm@unileon.es (J.F.G.M.); 2 School of Biosciences and Veterinary Medicine, University of Camerino, Via Circonvallazione 93/95, 62024 Matelica (MC), Italy; vincenzo.cuteri@unicam.it (V.C.); giacomo.rossi@unicam.it (G.R.); 3 Micros Veterinaria, INDEGSAL, via Profesor Pedro Carmenes s/n Campus de Vegazana, 24071 León, Spain; laurapolledo@gmail.com

**Keywords:** sheep, Small Ruminant Lentivirus, genetic characterization, udder, histopathology

## Abstract

Small Ruminant Lentiviruses (SRLVs) are widespread in many countries and cause economically relevant, slow, and persistent diseases in sheep and goats. Monitoring the genetic diversity of SRLVs is useful to improve the diagnostic tools used in the eradication programs. In this study, SRLVs detected in Spanish Assaf sheep with different grades of lymphoproliferative mastitis were sequenced. Genetic characterization showed that most samples belonged to type A and were closer to Spanish SRLV isolates previously classified as A2/A3. Four samples belonged to subtype B2 and showed higher homology with Italian B2 strains than with Spanish B2 isolates. Amino acid sequences of immuno-dominant epitopes in the gag region were very conserved while more alterations were found in the LTR sequences. No significant correlations were found between grades of mastitis and alterations in the sequences although samples with similar histological features were phylogenetically closer to each other. Broader genetic characterization surveys in samples with different grades of SRLV-lesions are required for evaluating potential correlations between SRLV sequences and the severity of diseases.

## 1. Introduction

Small Ruminant Lentiviruses (SRLVs) include *Visna-maedi virus* (VMV) and *Caprine arthritis encephalitis virus* (CAEV), which cause slow inflammatory diseases in sheep and goats named, respectively, Visna-Maedi (VM) and Caprine Arthritis Encephalitis (CAE). Viruses are transmitted mainly via the respiratory route and by colostrum intake [1,2] and cause persistent infections with a long incubation period. According to the current nomenclature based on *gag–pol* and *pol* sequences, SRLV can be subdivided into genotypes A–E with subtypes present in A, B, and E [3,4,5]. Typically, VMV was believed to infect specifically sheep and was included in genotype A while CAEV was considered goat-specific and was included in genotype B. However, several investigations showed that cross-infection may occur ([6] and other studies reviewed in [5]). Infected animals can develop neurological, pulmonary, arthritic, and/or mammary diseases that affect considerable animal welfare and production. Different patterns of inflammatory mononuclear cell accumulation are observed usually in the central nervous system, lung, joint, and/or udder and the predominant clinical manifestation depends on the severity and extension of the lesions reached in the affected organs [7]. Mammary lesions often consist of mononuclear cell infiltration with scattered hyperplastic lymphoid follicles [7,8]. Moderate lesions are described when diffused infiltration of lymphocytes within lobules with distortion of acini are observed and mild lesions are reported when occasional aggregates of lymphocytes in inter-acinar stroma are present [9]. Recently, minimal lesions consisting of a few small clusters to multifocal small groups of inflammatory cells with the presence of a few small lymphoid aggregates and/or one small lymphoid follicle have been reported in SRLV infected sheep [10].

One of the most important productive impacts of SRLV disease is due to the premature removal of diseased animals because of low milk production and quality with consequent economic losses in the milk-related and lamb/kids-related industry [11,12,13]. Due to the significant economic impact of diseases, VM and CAE are included in the OIE List and specific control and eradication programs are carried out in many countries [14,15]. Availability of sensitive and specific diagnostic tests is of great importance for a correct discrimination between infected and non-infected animals. Due to the high rate of genetic diversity, new genotypes and subtypes might escape the diagnostic detection with the possible consequence of invalidating any eradication program in place [1]. Therefore, genotype and subtype surveys of the circulating SRLVs should be encouraged. Many studies describe phylogenetic analysis of SRLVs found during epidemiological surveys or in outbreaks of diseases, but only a few of them describe the histopathological lesions observed in target organs [16,17,18]. For example, mild mammary lesions with a multifocal method to diffuse mononuclear inflammatory interstitial infiltrates have been observed in sheep with arthritis and infected by B2 SRLV [17]. Different histopathological scores have been reported in mammary glands but not in lungs, synovial membranes of joints, or the chorioid plexus of five goats infected by A4 SRLV [16]. A 13–14 nucleotide deletion in the R region of the LTR has been observed in sheep with a decreased pathology in the lung but not in the udder even though SRLV subgenotypes were not known [18]. To our knowledge, correlations among histopathological grading of mammary lesions and SRLV genotypes and subtypes are yet to be investigated. 

The aim of this work was to carry out genetic characterization and phylogenetic analysis of SRLV detected in Spanish sheep showing different histopathological grades of mastitis.

## 2. Material and Methods

### 2.1. Samples

A total of 35 udder samples were collected randomly at the slaughterhouse in the region of Castilla y Leon, Northwestern Spain, from Assaf sheep (1–4 years of age) belonging to seropositive flocks between March 2017 and May 2017. Nineteen samples were collected at the slaughterhouse named M (samples M1–M19) and 16 were collected at the slaughterhouse named Q (samples Q1–Q16), which was about 45 Km far from the slaughterhouse M. A first aliquot of each sample was stored at −20 °C and DNA was obtained from 25 mg of each sample by using the Genomic DNA isolation Kit (Norgen Biotek Corp., Thorold, ON, Canada) and following the manufacturer’s instructions when eluting the DNA in 100 µL final volume. A second aliquot of samples was fixed in 10% neutral buffered formalin for 48 h at room temperature and embedded in paraffin wax (FFPE) for histopathology and immunohistochemistry (IHC). In addition, 2 FFPE mammary samples (N16-426 and N-17-44) of sheep (Assaf breed, 3 years old) with histological mastitis referable to SRLV disease were available for this study. DNA from these latter samples was obtained from 4 slides 10 µm thick of each sample by Recover All Total Nucleic Acid Isolation (Ambion, Waltham, MA, USA) following the instructions and eluting DNA with 60 µL of elution solution warmed up at 95 °C.

### 2.2. Histopathology and Immunohistochemistry

Slides 4 µm thick were obtained from FFPE samples and were stained with haematoxylin and eosin (HE) for histopathology. Grading of histopathological lesions of mammary glands was carried out by three independent pathologists, which was previously described [10]. Briefly, “no lesions” was defined when no inflammatory cells were observed and “minimal lesions” (+) consisted of a few small clusters.

Multifocal small groups of inflammatory cells with the presence of a few small lymphoid aggregates and/or one small lymphoid follicle known as ‘moderate lesions’ (++) were characterized by the multifocal method to diffuse interstitial non-supportive inflammation and/or the presence of two to 15 lymphoid aggregates/follicles and ‘severe lesions’ (+++) consisted of a marked diffuse interstitial mastitis and/or the presence of >15 lymphoid aggregates/follicles. When differences between the severity of interstitial inflammatory infiltrates and the presence of lymphoid follicles were observed in the same organ of an animal, the most severe lesion was considered as the score for the lesion in that target organ [10].

Serial sections (4 µm) were used for IHC, which was previously reported [19]. A monoclonal antibody to the SRLV core protein p28 (VMRD Inc., Pullman, WA, USA) diluted 1:1000 was used. A technique based on an avidin-biotin-peroxidase complex (VECTASTAIN^®^ ELITE^®^ ABC Kits, Vector Laboratories, Burlingame, CA, USA) with diaminobenzidine as the chromogen (DAB Peroxidase substrate kit—Vector Laboratories, Burlingame, CA, USA) was used to stain the antigen.

### 2.3. PCR

A nested PCR was used to amplify about 800 bp long sequences of SRLV *gag-pol* genes, which was reported previously [4]. Primers GAG-F1 and POL-R1 were used in the first PCR. The product obtained was used as a template in a second PCR with primers GAG-F2 and POL-R2. The PCR mixture included 50 µL 2× Taq PCR Master Mix, 500 nM each primer, 4 µL DNA, and PCR grade water up to 100 µL final volume. PCR conditions were 94 °C for 5 min, 45 cycles of 94 °C for 1 min, 55 °C for 1 min, 72 °C for 2 min, and a final extension of 72 °C for 10 min. The second PCR was carried out with the same conditions but 5 µL of the first PCR products were used as the template and the annealing temperature was 60 °C instead of 55 °C [4]. LTR sequences (203 bp long) were amplified by nested PCR with primers described elsewhere [20]. The PCR reaction mix was described above, but 2 µL of DNA (first PCR) or 2 µL of the first PCR products (second PCR) were used as the template. PCR conditions were 94 °C for 5 min, 35 cycles of 94 °C for 30 s, 55 °C (or 50 °C in the second PCR) for 30 s, 72 °C for 40 s, and a final extension of 72 °C for 7 min. PCR products were visualized in 1.5% agarose gel and positive samples were submitted to an external laboratory for sequencing (BMR Genomics, Padova, Italy). Both the sense and antisense strands were sequenced by performing two independent reactions for each PCR product. Nucleotide *gag-pol* sequences were deposited in GenBank (Accession numbers MH179145—MH179153 and MH179156—MH179159).

### 2.4. Sequence Analysis

**Figure 1:**
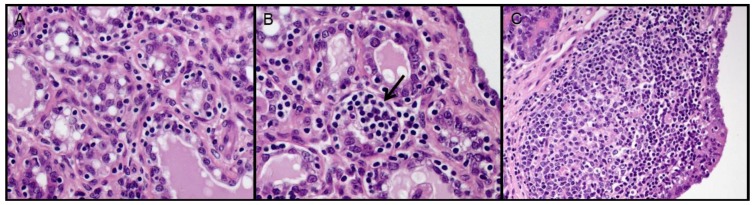
SRLV lesions in mammary gland of sheep. (**A**) Minimal lesion (+) with small focal lymphocyte aggregates within the mammary interstitium. 40×. (**B**) Black arrow indicates moderate (++) focal inflammatory lesion surrounded by minimal lymphocytic infiltrates. 40×. (**C**) Large lymphoid follicle in a severe lesion (+++). Hematoxylin and eosin (HE) staining. 20×.

Nucleotide sequences were manually checked and edited with the program BioEdit. A preliminary analysis by BLASTn was carried out to detect regions of similarity with sequences included in databases. Sequences of strains considered to be prototypes of different genotypes and SRLV sequences highly similar to those found in the samples were included in the study (Figure 1 and Figure 3). Sequences were aligned by MUSCLE [21] and phylogenetic trees were inferred with the program MEGA 7.0.21 [22]. The best-fitting nucleotide substitution models were estimated and the General Time Reversible model [23] with a gamma distribution with invariant sites (*gag* sequences) or a Kimura 2-parameter (LTR sequences) model [24] with gamma-distributed rates among sites were used with bootstrap values based on 1000 repetitions. Phylogeny was estimated by both the neighbor-joining algorithm (NJ) and the maximum likelihood (ML) method. Correlations among sequence alterations and histological features were evaluated by using the Fisher’s exact test. Pairwise distances between sequences of samples and sequences of reference strains belonging to different genotypes were calculated by MEGA 7.0.21 with the p-distance model [22].

## 3. Results

Histological examination and grading of mammary lesions (see Figure 1) resulted in five samples with severe lesions, 13 samples with moderate lesions, 11 samples with minimal lesions, and five samples without lesions (see Table 1). Grading of M5 sample was not possible due to a concomitant purulent mastitis. 

**Figure 2:**
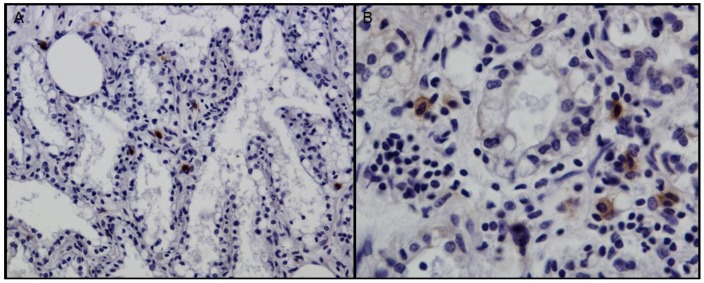
IHC against p28 of CAEV/VMV in mammary gland. (**A**) Scattered positive cells within minimal inflammatory lesions (+). 20×. (**B**) Positive macrophage-like cell in moderate lesion (++). 40×.

IHC results were used to distinguish SRLV infected from uninfected sheep. Three out of the five samples without lesions (M13, M18 and Q2) were negative by IHC and by both *gag-pol* and LTR PCR. Therefore, they were considered negative (see Table 1). The remaining 32 samples of groups M and Q were positive by IHC (see Figure 2). Sixteen out of the 32 M and Q samples and the 2 N samples were positive by *gag-pol* PCR, but good-quality sequences were obtained only from 15 samples. LTR PCR products were obtained in all but two IHC-positive samples.

### 3.1. Analysis of Gag Sequences

**Figure 3:**
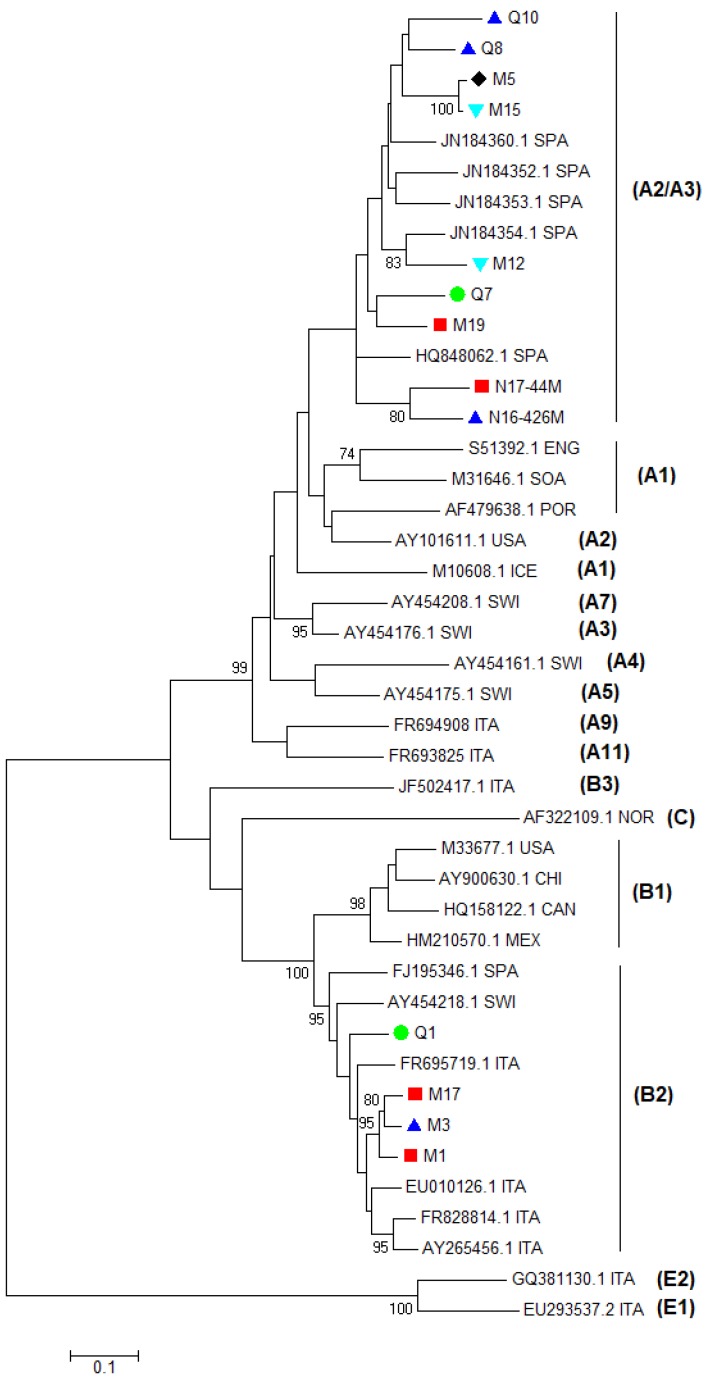
Phylogenetic analysis of the SRLV partial *gag-pol* region. Sequences of different SRLV genotypes and subtypes available in GenBank were used as reference isolates. Reference sequences are indicated with their accession number and country of origin (CAN: Canada; CHI: China; ENG: England; ICE: Iceland; ITA: Italy; MEX: Mexico; NOR: Norway; POR: Portugal; SOA: South Africa; SPA: Spain; SWI: Switzerland; USA: the U.S.A.). Samples are indicated with their codes and are labeled on the basis of the score of the mammary lesions observed (▲ severe, ■ moderate, ▼ minimal, ● no lesions, ♦ not available). The evolutionary history was inferred by using the Maximum Likelihood method based on the General Time Reversible model with a gamma distribution with invariant sites and with bootstrap values based on 1000 repetitions. The tree is unrooted.

Genotyping was carried out by phylogenetic analysis of partial *gag* gene sequences, according to the taxonomic classification proposed by Shah et al. [3]. All sequences were different from each other and nine samples were type A and four samples were type B. In particular, samples M5, M12, M15, M19, Q7, Q8, Q10, N16-426M, and N17-44M clustered within genotype A were more closely related with strains 292, 160, 166, and 697, which were previously detected in the same Spanish region (see Figure 3). Only strain 697 had been fully sequenced. Since similar values located with this isolate intermingled between A2 and A3, the isolate 697 had been assigned to the A2/A3 subtype [25]. Samples M1, M3, M17, and Q1 resulted of genotype B and subtype B2 (see Figure 3). Additionally, phylogenic and BLAST analysis showed that they were more related to B2 viruses detected in Italy than in Spain (Ov496). These results were confirmed by the pairwise distances comparison (see Table 2). Samples M12, M15, M19, Q7, Q8, Q10, N16-426, and N17-44 were more closely related to the A2/A3 Spanish strain HQ848062.1 (0.105–0.142). Moreover, samples M1, M3, M17, and Q1 were more closely related to the B2 strains FJ195346.1 and EU010126.1. In particular, they were more closely related to the Italian strain EU010126.1 (0.064–0.081) than to the Spanish strain FJ195346.1 (0.092–0.102).

**Figure 4:**
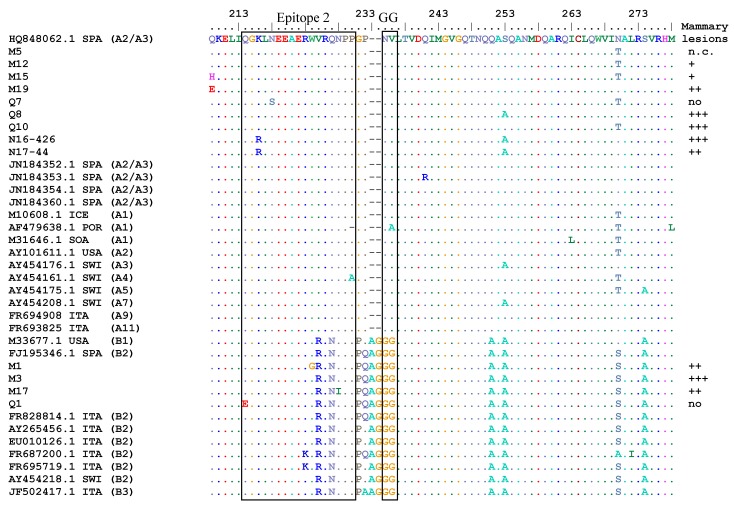

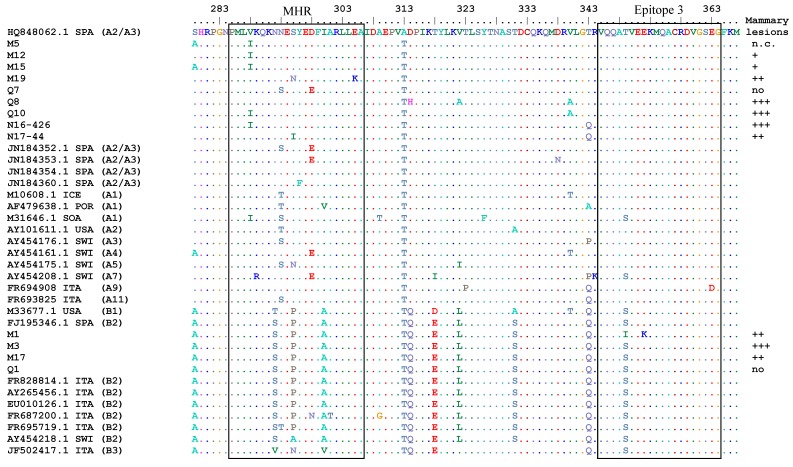
Alignment (MUSCLE) of deduced amino acid sequences of partial *gag*-p25 of the SRLV sequences obtained and of some SRLV reference strains; (**a**) positions from 209 to 278, (**b**) posiions from 279 to 368. Two immuno-dominant epitopes of this capsid protein, the GG motif, and the Major Homology Region (MHR) are within squares. The score of mammary lesion of each sample is reported. Legend: (·) homology, (−) deletion, (+++) severe lesions, (++) moderate lesions, (+) minimal lesions, (no) no lesions, (n. c.) not classified, CAN: Canada; CHI: China; ENG: England; ICE: Iceland; ITA: Italy; MEX: Mexico; NOR: Norway; POR: Portugal; SOA: South Africa; SPA: Spain; SWI: Switzerland; USA.

Nucleotide sequences were translated into amino acid sequences and the results of the alignment and comparison with the most representative sequences are reported in Figure 4a,b. The set of primers used in this study amplifies a partial sequence of the *gag* gene codifying for the majority of the capsid protein (CA). Comparing amino acid sequence alterations of the partial gag protein obtained, the “GG” motifs of the four type B sequences were glycine-glycine (GG) like type B reference SRLVs while those of the 11 type A samples were asparagine-valine (NV) like other type A reference SRLVs (see Figure 4a). In type A samples, sequences of epitopes 2 and 3 of reference isolates and of most samples were conserved (see Figure 4a). Only arginine (R) replaced lysine (K) in samples N16-426 and N17-44 and serine (S) replaced asparagine (N) in sample Q7. Type B samples had highly conserved epitope 3 sequences since there are only two alterations in M1 (isoleucine (I) instead of serine (S) and lysine (K) instead of glutamic acid (E). More alterations were found in epitope 2 where three out of four type B samples showed one alteration in comparison with type B reference isolates (see Figure 4a,b).

In the Major Homology Region (MHR), which is usually a highly conserved sequence in the *gag* gene of all retroviruses, some alterations were present in type A samples. In particular, all but one type A samples showed one or two alterations comparing to the A2/A3 reference strain 697 (see Figure 4b). Samples M5, M12, M15, Q10, and N16-426 had isoleucine (I) instead of valine (V) at the fourth position as type A1 reference strain SA-OMVV. This latter had also a serine (S) instead of asparagine (N) at the ninth position. Sample Q7 had not only this alteration, but also glutamic acid (E) instead of aspartic acid (D) at the 14th position, which shows the same alterations found in the A2/A3 Spanish strain 160. Sample N17-44 had isoleucine (I) instead of serine (S) at the 11th position unlike the other samples and reference strains. Unusual alterations were found in sample M19 where asparagine (N) replaced serine (S) at the 11th position and lysine (K) substituted glutamic acid (E) at the 21st position. Type B samples had highly conserved MHR sequences and showed the same amino-acidic sequences of B2 reference isolates even though some single alterations were present in the nucleotide sequences (see Figure S1). A significant correlation among sequence alterations and severity of mastitis was not found (*p* > 0.05).

### 3.2. Analysis of LTR Sequences

**Figure 5:**
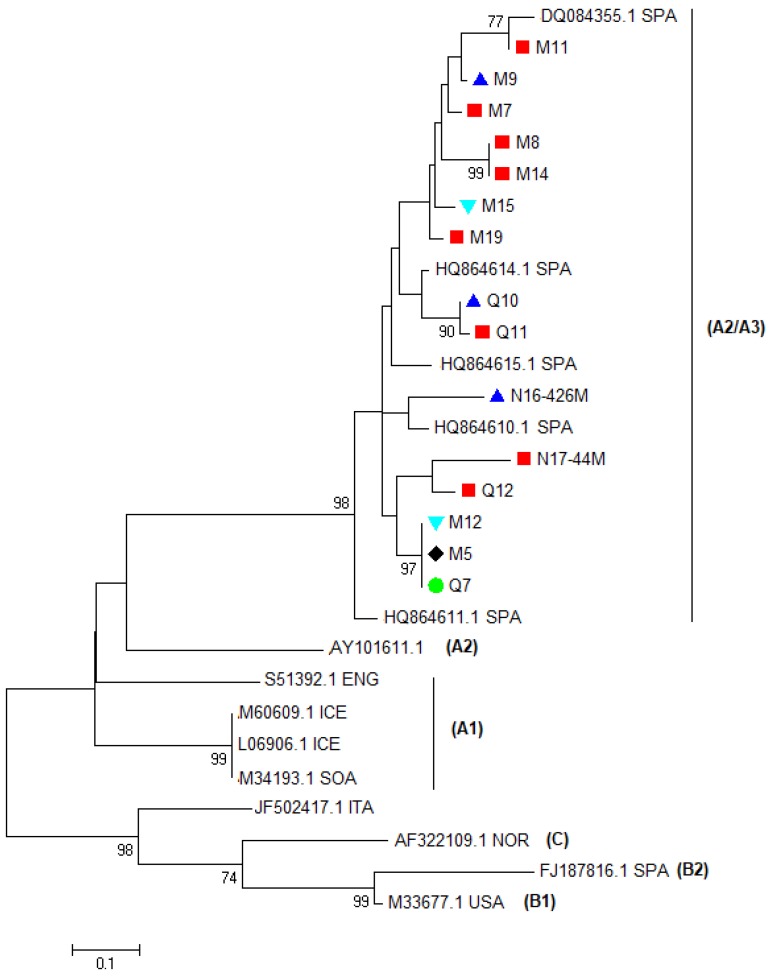
Phylogenetic analysis of the SRLV partial LTR. Sequences of different SRLV genotypes and subtypes available in GenBank were used as reference isolates. Reference sequences are indicated with their accession number and country of origin (CAN: Canada; CHI: China; ENG: England; ICE: Iceland; ITA: Italy; MEX: Mexico; NOR: Norway; POR: Portugal; SOA: South Africa; SPA: Spain; SWI: Switzerland; USA). Samples are indicated with their codes and are labeled on the basis of the score of the mammary lesions observed (▲ severe, ■ moderate, ▼ minimal, ● no lesions, ♦ not available). The phylogenetic analysis was performed with a maximum likelihood (ML) method using the Kimura 2-parameter model with a gamma distribution and with bootstrap values based on 1000 repetitions. Sequences are not deposited in GenBank because they do not reach the minimum length required.

**Figure 6:**
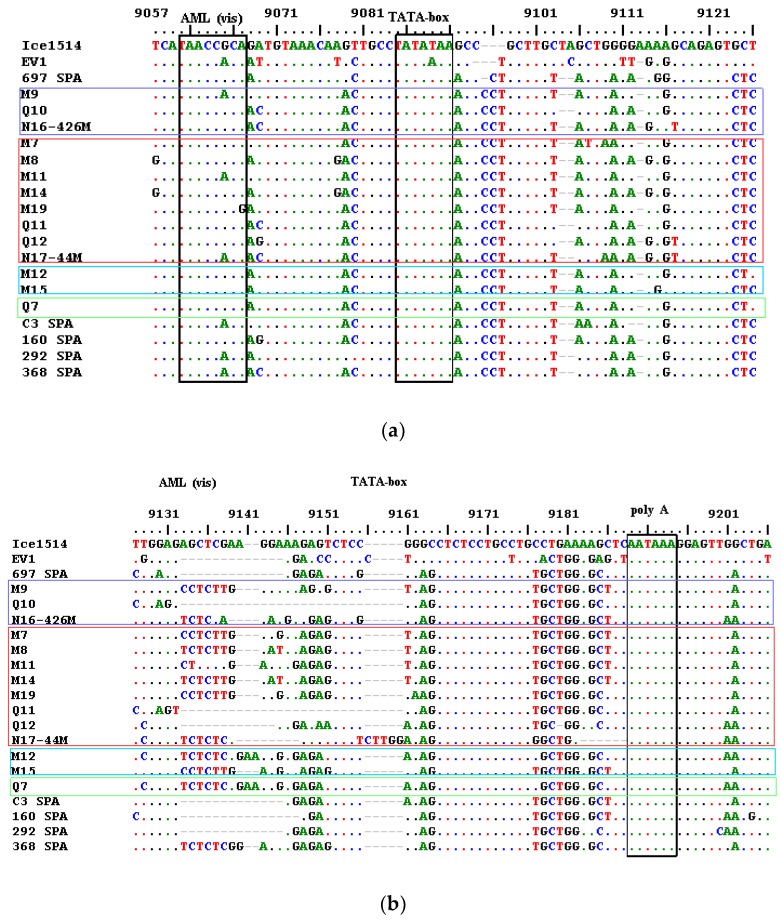
Alignment (MUSCLE) of nucleotide sequences of the LTR region of the SRLV sequences obtained and of some SRLV reference strains. LTR sequence of the isolate Icelandic 1514 (M10609.1) was used as a reference; (**a**) positions from 9057 to 9126, (**b**) posiions from 9127 to 9206. Sequence of reference strains EV1 (S51392.1) was also used. Sequences previously found in Spain in goats (C3—DQ084355.1) and in sheep (697—HQ864615.1, 160—HQ864610.1, 292—HQ864611.1 and 368—HQ864614.1) and showing high homology with our samples were also included. Sequences of the AML (vis), the TATA box element, and the polyadenylation signal are within black squares. Sequences of samples with similar scores of mammary lesions are within colored squares. Legend: (·) homology, (−) deletion, (blue squares) severe lesions, (red squares) moderate lesions, (light blue squares) minimal lesions, and (green squares) no lesions.

The alignment and phylogenetic analysis of LTR nucleotide sequences showed that most samples were closer to the reference Spanish A2/A3 strain 697 (Figure 5 and Figure 6a,b). Comparing samples with the 697 reference strain, Q10 and Q11 showed a 23 nt deletion (9133–9160 nt) in the R region, which appeared longer than the 13 nt deletion present in the reference strains 697, EV1, and in other Spanish strains (C3, 160, 292). Sample Q12 had similar deletions than 697 while other samples showed 2–8 nt deletions in the same tract. No significant differences were found among sequences of samples with a different grade of mastitis (*p* > 0.05). Similarly, as previously described, the TATAbox, the polyadenylation signal, and the AML (vis), which is a site possessing the consensus sequence for the AML/PEBP2/CBF transcriptional factor family [26], were conserved and only a substitution G with A was present in three samples at position 9065 of AML (vis).

## 4. Discussion

This study describes for the first time *gag* and LTR sequences of SRLVs detected in Spanish Assaf sheep with different grades of histopathological mastitis and their phylogenetic relationships in the context of known SRLV sequences. 

Although initially genotypes B viruses were thought to infect only goats, it is not unusual to find reports about infections by type B viruses in sheep and by type A viruses in goats [27,28,29,30]. Even in this study, both genotypes A and B SRLVs have been found in ovine samples. It was not known if the examined sheep had contact with goats, but infection with A genotypes in goats was not reared in contact with sheep, which was reported [31].

The *gag-pol* phylogenetic tree and the pairwise genetic distances comparison revealed that most sequences of the samples were closer to the Spanish A2/A3 isolate 697 while four sequences belonged to subtype B2. Isolate 697 has been previously detected in sheep with neurological diseases from the Spanish region of Castilla y León in Spain and has been classified as A2/A3 because differences between A2 and A3 are often not large enough to separate the two groups [3,25]. Partial sequences of viruses classified as A2/A3 have been detected further seven sheep with neurological signs in Spain [25]. These findings suggest that, in Northern Spain, subtype A2/A3 SRLVs is genetically related to SRLVs, which caused nervous diseases. However, in our cases, neurological signs were not reported. Samples M1, M3, M17, and Q1 were closer to Italian than to Spanish B2 isolates and mammary lesions were found from moderate to severe in three out of four samples while, in sample Q1, lesions were not found. B2 SRLV has been detected in Spain for the first time in SRLV-seropositive adult sheep of the Rasa Aragonesa breed, which shows clinical signs of arthritis [17,32]. Mammary histological lesions were present in 10 out of 13 animals with arthritis, which suggests that udders can be involved even if clinical signs might remain unrecognized until the losses of milk production are significant. B2 viruses have been detected in Italian small ruminants during epidemiological surveys, but data about clinical signs or histological lesions are not reported [4,33].

In addition, good-quality sequences about 800 bp long were obtained from FFPE samples. Fixation in formalin and embedding in paraffin at high temperatures is thought to degrade DNA. Fragmentation of DNA molecules can interfere with their amplification by PCR and with consequent sequencing. In our case, good-quality DNA has been extracted and amplified by PCR from archival FFPE samples, which suggests that this method could be attempted for studying FFPE samples as well as for retrospective investigations.

Analysis of the genetic sequences is important not only for evaluating the spread of SRLV types and subtypes but also for monitoring antigenic variability. Actually, remarkable antigenic variation might be responsible for the misdiagnosis of highly divergent genotypes [34]. The *gag* gene encodes nucleocapsid, capsid, and matrix proteins. Indirect diagnostic assays usually use the capsid protein as the antigen, which helps monitor immuno-dominant epitopes of *gag*-encoded structural proteins. This is useful for detecting antigenic variability in the field and forevaluating and improving the sensitivity of indirect diagnostic tools. Alterations in the amino acid sequences of immuno-dominant epitope regions suggest altered antigenicity, which may affect the sensitivity of serological tests such as ELISA and AGID. The *gag-pol* set of primers used in this study allowed sequencing only of epitopes 2 and 3. Amino acid sequences of epitopes 2 and 3 of type A2/A3 viruses were quite conserved and limited alterations only in three and one samples, respectively. Epitope 2 of B2 isolates had more alterations, which shows single amino acid alterations in three out of four sequences. In addition, more variability was found in the MHR of A2/A3 viruses, which show all but two samples and at least one alteration in comparison with the reference A2/A3 strain 697. The MHR is usually conserved in many retroviruses and is essential for viral assembly [35]. Mutations in the MHR sequence of HIV-1 cause capsid assembly that reduces infectivity [36]. While some studies have been carried out on MHR of human retroviruses, the consequences of MHR mutations on infectivity of SRLVs should be better investigated. MHR of B2 viruses and GG motif of both A2/A3 and B2 viruses in the gag amino acid sequences, AML (vis) motif, TATA-box, and poly-A of both A2/A3 and B2 viruses in the LTR nucleotide sequences were highly conserved, which was previously reported in strains from different geographic areas [31,37,38].

Most LTR sequences showed higher homology with A2/A3 Spanish SRLV isolates. Samples Q10 and Q11 showed a 23 nt deletion in the R region, which appeared longer than the 13 nt deletion observed in type A2/3 reference isolate 697. A 13 nt deletion in this region has been found in sequences of clinically affected sheep and a correlation among this deletion and the appearance of clinical signs has been suggested [32]. On the contrary, a similar deletion has been found in SRLVs infecting asymptomatic sheep and the lungs of animals infected with viruses carrying the deletion were significantly less affected than sheep infected with viruses without deletion [18]. In the present study, significant correlations among deletions in the R region of the LTR and severity of mammary lesions were not found (*p* > 0.05). Samples with deletions were from sheep with more severe mammary lesions and sheep with moderate to severe mastitis did not show this deletion (see Figure 6b).

Although the severity of mammary lesions was not significantly related to the viral genotype, SRLV sequences from samples with similar grades of lesions (no/minimal and moderate/severe) were most closely related to each other (see Figure 3 and Figure 5 and Table 2). Considering the high economic impact of SRLV diseases, some countries aim to eradicate the diseases by identifying and prematurely culling infected animals. Selecting animals on the basis of serological results could determine the selection of SRLV variants with significant alterations in the antigen sequences. Permanent and extensive surveys should be encouraged in different countries to evaluate the antigenic variability of SRLV and to monitor the sensitivity and specificity of diagnostic tests in detecting these variants. In particular, seronegative animals should be investigated for infections by new viral genotypes not detected by traditional serological tests. Histological screening of different target organs at the slaughterhouse could be a useful tool for selecting samples with lesions, which suggests an SRLV disease in seronegative animals.

## 5. Conclusions

In conclusion, this is the first study investigating the association between the SRLV sequence analysis and histopathological grading of mammary lesions in sheep. Circulation of SRLVs of types A2/A3 and B2 in Spanish Assaf sheep was confirmed and new viral variants have not been found, but moderate alterations were present in some immuno-dominant epitopes and in the MHR tract.

No significant correlation was found among histological features and alterations in the sequences. Although some sequences obtained from samples with similar grades of mammary lesions appeared closer to each other, more extensive and interdisciplinary studies are required for establishing the existence of viral clusters with a higher or lower pathogenicity for specific target organs.

## Figures and Tables

**Table 1:** List of ovine mammary gland samples collected for this study. Samples are classified on the basis of the grade of mastitis observed by histopathology. Lesions in sample M5 were not classified due to concomitant purulent mastitis was present. “+”: positive result, “−”: negative result.

Sample	Grade of Mastitis	IHC	LTR PCR	*Gag-pol* PCR	Genotype
M1	moderate	+	+	+	B2
M2	minimal	+	+	−	−
M3	severe	+	+	+	B2
M4	moderate	+	+	−	−
M5	not classified	+	+	+	A2/A3
M6	moderate	+	+	−	−
M7	moderate	+	+	+	−
M8	moderate	+	+	+	−
M9	severe	+	+	+	−
M10	minimal	+	+	−	−
M11	moderate	+	+	+	−
M12	minimal	+	+	+	A2/A3
M13	no	−	−	−	−
M14	moderate	+	+	−	−
M15	minimal	+	+	+	A2/A3
M16	minimal	+	+	−	−
M17	moderate	+	+	+	B2
M18	no	−	−	−	−
M19	moderate	+	+	+	A2/A3
Q1	no	+	+	+	B2
Q2	no	−	−	−	−
Q3	minimal	+	−	−	−
Q4	minimal	+	+	−	−
Q5	minimal	+	+	−	−
Q6	severe	+	+	−	−
Q7	no	+	+	+	A2/A3
Q8	severe	+	+	+	A2/A3
Q9	moderate	+	+	−	−
Q10	severe	+	+	+	A2/A3
Q11	moderate	+	+	−	−
Q12	moderate	+	+	+	−
Q13	minimal	+	−	−	−
Q14	moderate	+	+	−	−
Q15	minimal	+	+	−	−
Q16	minimal	+	+	−	−
N16-426	severe	+	+	+	A2/A3
N17-44	moderate	+	+	+	A2/A3

**Table 2:** Pairwise nucleotidic genetic distances (p-distance model) of the partial *gag-pol* region of some SRLV reference strains and SRLV strains sequenced in this study.

Sample	Genotype	M5	M12	M15	M19	Q7	Q8	Q10	N16-426	N17-44	M1	M3	M17	Q1
M12		0.112	-											
M15		0.013	0.013	-										
M19		0.013	0.122	0.104	-									
Q7		0.130	0.133	0.129	0.109	-								
Q8		0.100	0.115	0.097	0.122	0.122	-							
Q10		0.109	0.119	0.105	0.129	0.127	0.105	-						
N16-426		0.127	0.138	0.129	0.115	0.137	0.127	0.135	-					
N17-44		0.152	0.155	0.152	0.138	0.163	0.157	0.152	0.094	-				
M1		0.208	0.213	0.208	0.213	0.216	0.209	0.216	0.217	0.216	-			
M3		0.216	0.222	0.211	0.221	0.231	0.211	0.221	0.224	0.217	0.040	-		
M17		0.214	0.221	0.209	0.226	0.232	0.217	0.217	0.231	0.227	0.048	0.041	-	
Q1		0.209	0.216	0.203	0.217	0.222	0.221	0.219	0.216	0.216	0.081	0.086	0.076	-
M10608.1	A1	0.171	0.160	0.168	0.137	0.157	0.166	0.145	0.175	0.175	0.214	0.208	0.229	0.224
S51392	A1	0.173	0.165	0.168	0.173	0.181	0.171	0.163	0.176	0.168	0.242	0.247	0.245	0.244
AY101611.1	A2	0.155	0.135	0.153	0.150	0.147	0.153	0.152	0.165	0.165	0.186	0.188	0.191	0.201
HQ848062.1	A2/A3	0.117	0.135	0.120	0.105	0.122	0.127	0.135	0.142	0.124	0.206	0.209	0.219	0.213
AY454176.1	A3	0.130	0.138	0.130	0.130	0.137	0.133	0.145	0.145	0.152	0.178	0.191	0.193	0.198
AY454161.1	A4	0.138	0.166	0.135	0.153	0.185	0.143	0.150	0.173	0.171	0.211	0.216	0.214	0.214
AY454175.1	A5	0.153	0.145	0.155	0.148	0.171	0.160	0.147	0.176	0.165	0.209	0.203	0.208	0.206
AY454208.1	A7	0.176	0.157	0.170	0.145	0.158	0.158	0.166	0.161	0.140	0.198	0.209	0.201	0.209
FR694908	A9	0.166	0.168	0.170	0.166	0.178	0.180	0.171	0.181	0.161	0.213	0.224	0.224	0.231
FR693825	A11	0.183	0.171	0.180	0.152	0.157	0.176	0.183	0.189	0.178	0.213	0.217	0.231	0.232
M33677	B1	0.204	0.214	0.201	0.211	0.224	0.211	0.211	0.222	0.211	0.120	0.119	0.120	0.129
FJ195346.1	B2	0.226	0.231	0.221	0.214	0.232	0.226	0.237	0.221	0.217	0.092	0.092	0.102	0.100
EU010126.1	B2	0.208	0.222	0.206	0.226	0.232	0.219	0.227	0.224	0.216	0.064	0.068	0.068	0.081
JF502417.1	B3	0.213	0.221	0.214	0.209	0.217	0.219	0.232	0.213	0.224	0.185	0.186	0.196	0.178
AF322109.1	C	0.262	0.252	0.257	0.250	0.252	0.245	0.255	0.244	0.236	0.213	0.214	0.214	0.216
EU293537.2	E1	0.292	0.295	0.292	0.297	0.301	0.293	0.300	0.293	0.290	0.293	0.285	0.290	0.303
GQ381130.1	E2	0.290	0.301	0.293	0.290	0.313	0.293	0.298	0.288	0.290	0.290	0.290	0.297	0.293

## Supplementary Materials

The following are available online at http://www.mdpi.com/1999-4915/10/6/315/s1, Figure S1: Nucleotide alignment of MHR sequences in the *gag* gene of the samples and of some reference strains.
