# Supplementary material for: Genetic Characterization and Phylogenetic Analysis of Small Ruminant Lentiviruses Detected in Spanish Assaf Sheep with Different Mammary Lesions
Source: Viruses. 2018 Jun 9;10(6):315. doi: 10.3390/v10060315 (PMC6024768; doi:10.3390/v10060315)
Supplement: Supplementary file 1 [file viruses-10-00315-s001.pdf]

```
                                                        MHR
                                 1348      1358      1368      1378      1388      1398      1408
                                 ....|....|....|....|....|....|....|....|....|....|....|....|....|.
HQ848062.1_A2/A3_697_SPA         CCCATGTTAGTAAAGCAAAAGAACAATGAGAGTTATGAAGATTTTATAGCAAGACTGTTGGAAGCA
M5                               ..A......A...................A........G..............G.....A...... n.c.
M12                              ..T...C..A.......G...........A..C..................C........A...... +
M15                              ..A......A...................A........G..............G.....A...... +
M19                              ......C..........................A........C..............A..AA..... ++
Q7                               ......C..................G...............G............T.A..A...... no
Q8                               ..T...C......................A........G...............T....A...... +++
Q10                              ......C..A.............T.....A........G...............T.A..A...... +++
N16-426M                         ......C..A....A........T.....A..C.....G...............T....A.....G +++
N17-44M                          ......C.......A........T.......TC.....G........................... ++
JN184352.1_A2/A3_160_SPA         ..A...C..................G...............G............T.A.........
JN184353.1_A2/A3_166_gag_SPA     ......C.G..G....................C.....G..G...........GT...........
JN184354.1_A2/A3_292_SPA         .................G..............C.....G.....................A......
JN184360.1_N-05/136_SPA          ..A...C.G.................C.......T...................T...........
M10608.1_A1_Icelandic1514_IC     ..T........G.....G.....T.C...............C..C.....TC.C..AC.A..G..T
AF479638.1_A1_P1OLV_POR          ..T...C.G..............T.CA...........G..C...G....G........A......
M31646.1_A1_SA-OMVV_SOA          ..T...C.GA....A........T.G...A.......................T..C.A......
AY101611.1_A2_85/34_USA          ..T...C..........G..A..T.CA..............C..C.....G...............
AY454176.1_A3_SNCR5561_SWI       ..T........G........A..T.G...A.....................C..GT.A..A.....T
AY454161.1_A4_SNCR4668_SWI       ..T.....G........G..A..T.....A........G..A..C...........C..A......
AY454175.1_A5_SNCR5560_SWI       ..T...........A........T.G...A.AC.....G..C..C.....G..GT.A..A......
AY454208.1_A7_SNCR5692_SWI       ..T..........G.........T........C........G........C...T.A..A.....C
FR694908_A9_1017/UM/10_ITA       .................G..A..T........C...........C.....C..G..A.....G...
FR693825_A11_0245/UM/10_ITA      ..A...C.G........G.....T.G..................C.........T.A..A..G...
M33677.1_B1_CAEV-Co_USA          ..A...C.............A.CG......CCA............GC.........C.A......
FJ195346.1_B2_Ov496_SPA          ..T........G..A.......GT......CCA.......C...GC.......G...C.A..G...
JF502417.1_B3_Volterra_ITA       ..A...C....G.........GTA.....A.A......G..C...G.T..T...T.A........C
M1                               ..T...........A..G..A.GT......CCA............GC.........C....G... ++
M3                               ..T...........A.....A.GT......CCA............GC.........C....G... +++
M17                              ..T...........A.....A.GT......CCA............GC.........C......... ++
Q1                               ..T...........A.....A.GT......CCA.....G......GCG....G...C......... no
FR828814.1_B2_AN13/MA/10_ITA     ..T...........A.....A.GT......CCA............GC.....G...C.........
AY265456.1_B2_It-Pi1_ITA         ..T...........A.......GT......CCA............GCC....G...C.A......
EU010126.1_B2_It-007.5s03_IT     ..T........G..A.......G.......CCA............GC..G..G...C....G...
FR687200.1_B2_06/PI/09_ITA       ..T...C.......A.......GT......CCA......A.....GCCA...G...C....G...
FR695719.1_B2_7008/UM/10_ITA     ..T...........A.......GT.C....CCA............GC..........A......
AY454218.1_B2_SNCR5720_SWI       ..A.................A.G.......GCA.....G......GC.....G...C.A......
```
